# The emerging role of *Fusobacteria* in carcinogenesis

**DOI:** 10.1111/eci.14353

**Published:** 2024-12-14

**Authors:** Raisha J. Gibbs, Adam C. Chambers, Darryl J. Hill

**Affiliations:** ^1^ University of Bristol Bristol UK

**Keywords:** cancer, carcinogenesis, CEACAM, colorectal, *Fusobacteria*, signalling

## Abstract

The *Fusobacterium* genus comprises Gram‐negative, obligate anaerobic bacteria that typically reside in the periodontium of the oral cavity, gastrointestinal tract, and female genital tract. The association of Fusobacterial spp. with colorectal tumours is widely accepted, with further evidence that this pathogen may also be implicated in the development of other malignancies. Fusobacterial spp. influence malignant cell behaviours and the tumour microenvironment in various ways, which can be related to the multiple surface adhesins expressed. These adhesins include Fap2 (fibroblast‐activated protein 2), CpbF (CEACAM binding protein of *Fusobacteria*), FadA (*Fusobacterium* adhesin A) and FomA (Fusobacterial outer membrane protein A). This review outlines the influence of *Fusobacteria* in promoting cancer initiation and progression, impacts of therapeutic outcomes and discusses potential therapeutic interventions where appropriate.

## Graphical abstract

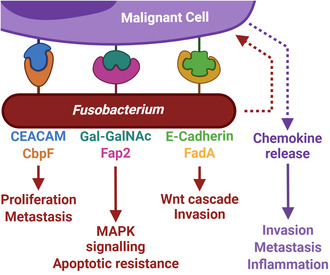

## 1 INTRODUCTION

The human microbiome is a complex ecosystem encompassing various microorganisms, including viruses, bacteria, and fungi, culminating in numerous host‐microbe relationships. The gastrointestinal tract contains the highest microbial diversity, wherein bacteria constitute the majority of microbes across a gradient from the ileum to the cecum of 10^8^ bacteria/g to 10^11^ bacteria/g.^1^ Microbe‐host interplay contributes to various pathologies, including tumorigenesis.^2^ Links between malignancies and viruses are well established, with the Epstein–Barr virus being associated with Burkitt's lymphoma,^3^ and the role of the human papillomavirus in cervical cancer being irrefutable.^4^ However, the role of the bacteria within the microbiome, termed microbiota, in carcinogenesis remains disputable and poorly categorised except for a few exceptions, such as the correlation between *Helicobacter pylori* infection and gastric cancers.^5^ Considering the vast diversity of the colonic microbiota, colorectal cancer (CRC) has been at the forefront of research concerning this dichotomous relationship. The first observation of bacterial presence in tumours is documented over a century ago,^6^ but the topic continues to garner significant attention. Several bacterial species have been correlated with CRC incidence, including but not limited to *H. pylori*, *Escherichia coli*, *Streptococcus gallolyticus*, *Bacteroides fragilis*, *Enterococcus faecalis* and, most prominently, *Fusobacteria* spp.^7^, ^8^, ^9^, ^10^, ^11^, ^12^, ^13^

**FIGURE 1:**
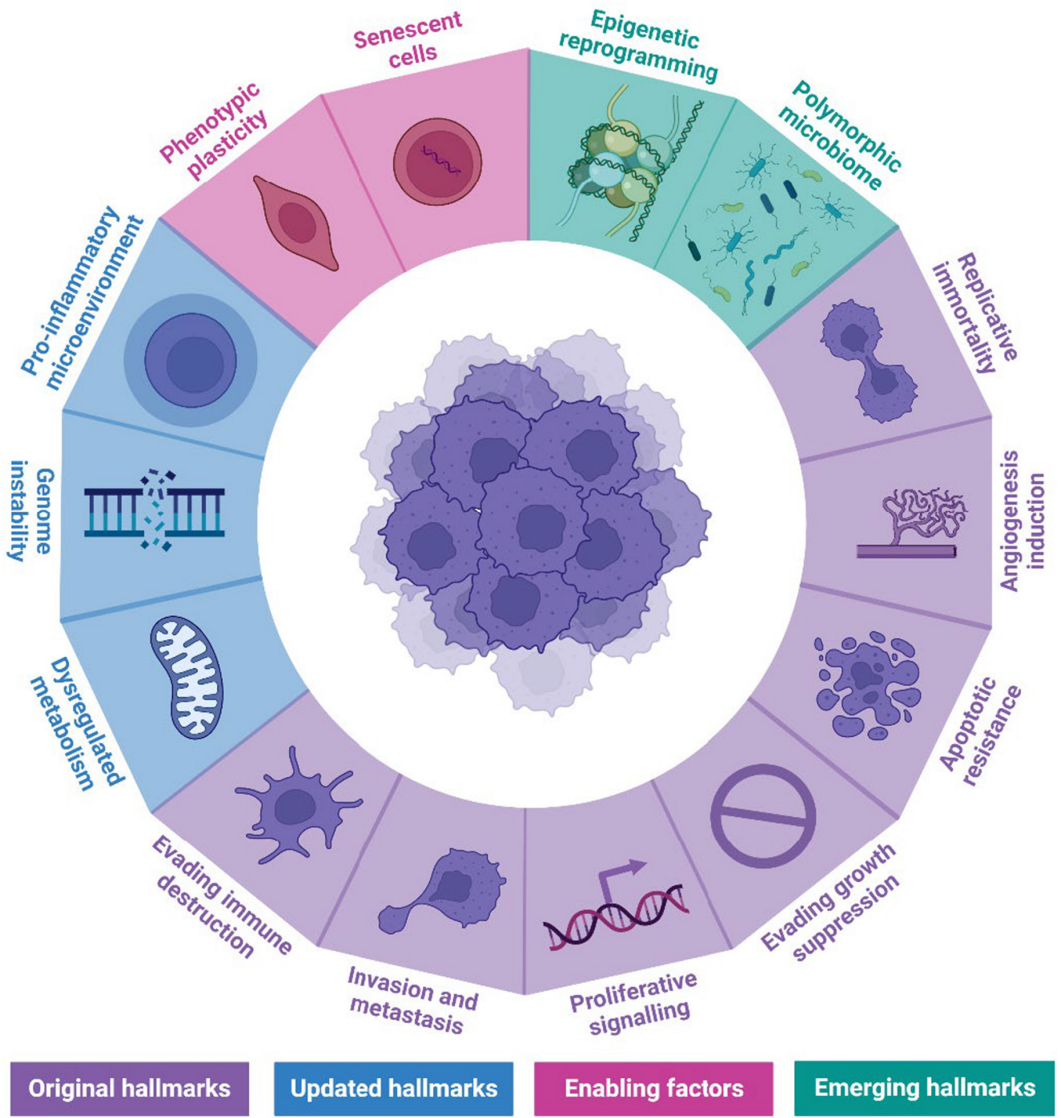
The hallmarks of cancer. The classical/original hallmarks of cancer (purple) have been expanded upon since their proposal in 2000. The hallmarks now consist of 14 distinct attributes. Created in BioRender and adapted from information in.^22^, ^23^, ^24^

*Fusobacteria* are Gram‐negative, non‐motile, obligate anaerobic bacteria^14^ known to colonise the periodontium, female genital tract and gastrointestinal tract.^15^ The *Fusobacteriaceae* family is further divided into the heterogenous genus of *Fusobacterium*, featuring a genome of 2.17 Mb with over 2000 open reading frames.^16^ Several distinct species of *Fusobacterium* exist, with *Fusobacterium nucleatum* (Fn) perhaps being the most extensively studied. Crucially, a large degree of population heterogeneity exists, with Fn exhibiting a pangenome of over 6600 gene clusters, 87% of which are accessory genes.^17^ *Fusobacteria* are associated with several pathologies, including chronic periodontitis and inflammatory bowel disease. *Fusobacteria* are also evidenced to be involved in the progression of CRC.^18^ Pangenomic analysis has identified 483 enriched clusters within *Fusobacterium* strains which are associated with CRC in comparison to orally associated strains.^19^ Of the *Fusobacterium* genus, Fn is the predominant species implicated in carcinogenesis, with the suggestion that two subspecies are also heavily involved in malignant transformation: *Fusobacterium nucleatum vincentii* and *Fusobacterium nucleatum animalis*.^20^ Importantly, genetic analysis has revealed two distinct clades of the *animalis* subspecies, with results suggesting that only the second clade (Fna‐C2) is enriched in CRC.^19^ Regardless, the influence of Fusobacterial strains on carcinogenic processes can be discussed concerning adhesins expressed in the outer membrane, principally Fap2 (Fibroblast‐activated protein‐2), CbpF (CEACAM binding protein of *Fusobacteria*), FadA (*Fusobacterium* adhesin A) and FomA (Fusobacterial outer membrane protein A).^21^ Interaction between *Fusobacteria* and malignant cells may enhance several of the classical cancer hallmarks (Figure 1),^18^ including replicative immortality, apoptotic resistance and immune evasion.^22^, ^25^

This review will explore the interplay between carcinogenesis and *Fusobacteria*, focusing on adhesins and other outer membrane proteins. Potential therapeutic targets directed against these outer membrane proteins will also be addressed. The role of *Fusobacteria* in disease progression and therapeutic resistance also be discussed.

## 2 CANCERS ASSOCIATED WITH *FUSOBACTERIA*

**FIGURE 2:**
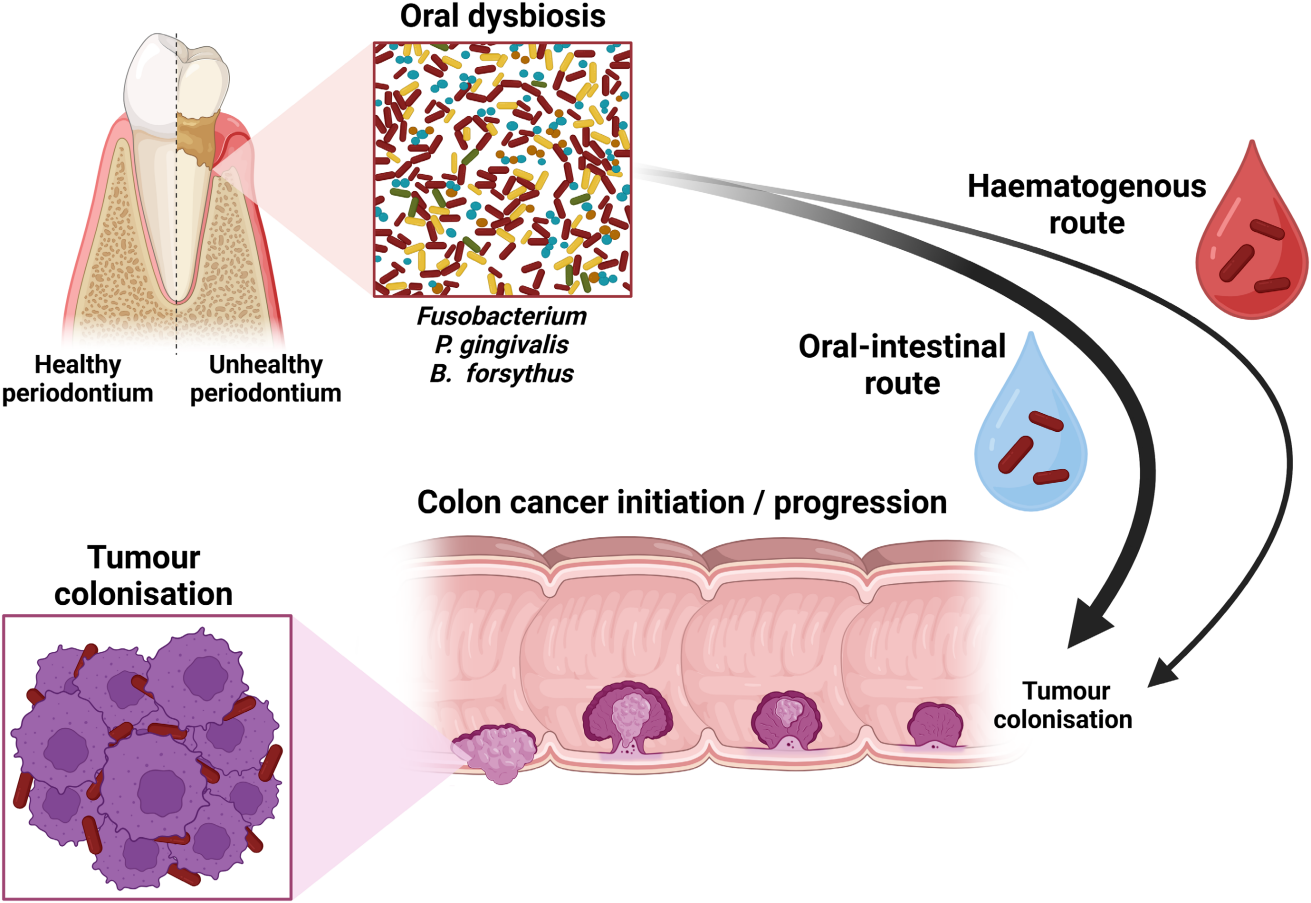
Depiction of *Fusobacteria* travelling to the gastrointestinal tract. In chronic periodontitis, oral dysbiosis is observed, with an increase in several bacterial species. The oral‐intestinal route represents the swallowing of *Fusobacteria*, be it individually or as part of a biofilm with mixed population. The haematogenous route, for example, with gingival bleeding, allows for the swallowing of blood containing *Fusobacteria*, allowing the transport of bacteria to the gastrointestinal tract. Here, *Fusobacteria* is able to promote malignant transformation, leading to the development and/or progression of colorectal cancer. Line and arrow weight is proportional to the frequency of routes used. Created in BioRender and adapted from information within.^27^

The first characterised association of *Fusobacteria* in CRC was reported in 2011, wherein tumour samples and adjacent non‐affected samples were analysed, revealing an enrichment of Gram‐negative bacteria in tumour tissues, with a subset displaying extremely high enrichment with 89% of bacterial DNA belonging to Fusobacterial species.^11^ Similarly, tumour samples also contain higher levels of Fusobacterial RNA compared to matched controls, suggesting a role for actively transcribing *Fusobacteria* in CRC.^26^ Now, CRC is the predominant cancer associated with fusobacterial colonisation. However, this likely reflects the lack of primary datasets investigating alternative malignancies.^18^ Regardless, in CRC, this opportunistic pathogen colonises the periodontium and travels to the gastrointestinal tract in two major ways. Primarily, the swallowing of plaques and biofilms containing *Fusobacteria*, which enables access to the tumour site and is termed the oral‐intestinal route. To a lesser extent, *Fusobacteria* can travel to tumour sites via haematogenous spread, wherein *Fusobacteria* enter and utilise the vasculature to ultimately colonise tumours^27^ (Figure 2). Residing in colorectal tumours, it is hypothesised that Fn promotes tumourigenic processes through various mechanisms.^28^ However, it remains unclear whether Fusobacterial tumour colonisation is an initiating event in CRC tumourigenesis or whether the malignancy provides the perfect niche to facilitate Fusobacterial colonisation.

### 2.1 Carcinogenesis

Cumulative research exists to support the role of Fusobacterial spp. in various malignancies, including gastric,^29^ pancreatic^30^ and breast^15^ cancer. Fusobacterial presence in tumour tissue samples is associated with several clinicopathological features across various malignancies.

Genetic abnormalities are well documented in CRC, including the loss‐of‐function mutation of the adenomatous polyposis coli (*APC*) gene, leading to constitutive activation of the Wnt/β‐catenin signalling pathway. Importantly, samples enriched with Fn display higher levels of *APC* mutations,^31^ potentially facilitating the accumulation of excess β‐catenin and ultimately increasing cellular proliferation and mitosis.^32^ Epigenetic abnormalities are also observed in Fn colonised tissues, with chromatin changes observed following exposure. Interestingly, this epigenomic restructuring largely depends on host cell type, with results differing between cell lines.^33^ Notably, microsatellite instability (MSI) is considerably higher in Fn‐colonised CRC.^34^ Furthermore, CpG methylation status (CIMP) is significantly altered by the presence of Fn in CRC samples. However, this notion is unsurprising given that a well‐recognised subset of CRC tumour type is CIMP high MSI.^35^ Regardless, Fusobacterial exposure triggers hypermethylation of the *CDKN2A* gene (cyclin‐dependent kinase inhibitor 2A), resulting in gene repression.^36^ Suppression of *CDKN2A* allows cell cycle progression, thereby allowing for CRC cell proliferation. Therefore, increased *CDKN2A* hypermethylation induced by Fn further contributes to the augmented proliferative signalling that is considered a major hallmark of cancer.^37^, ^38^, ^39^ Interestingly, no correlation exists in CRC between fusobacterial abundance and clinical features such as tumour stage or anatomical location. This may suggest that Fusobacterial positivity may serve as an independent prognostic factor for CRC patients.^40^

Augmented proliferation rates in response to Fn exposure have also been observed in the context of oesophageal cancer. In vitro, Fn increased proliferation, migration and invasive capacity in human oesophageal squamous cell carcinoma cell lines (TE‐8 and TE‐10).^41^ Upregulation of the NF‐κB signalling pathway, which culminates in the transcription of proinflammatory cytokines, was revealed to be the underpinning mechanism for these malignant cell behaviours.^41^ Moreover, the promotion of chemokine CCL20 by *Fusobacteria* ^42^ has been shown to work in axis with CC6 in oesophageal cancers to promote proliferation and migration, ultimately promoting cancer progression.^43^

### 2.2 Chemotherapeutic response

Dysbiosis is associated with the elevation of anaerobic bacterial species^28^ and alterations in therapeutic response. Chemoresistance can be induced by Fn in various malignant cells, with the suggestion that modulation of autophagy is likely the underpinning mechanism.^44^, ^45^, ^46^, ^47^ Regardless, Fusobacterial intratumoral levels are intimately correlated with therapeutic responses in multiple cancers. High levels of Fn in oesophageal squamous cell carcinoma is associated with poor recurrence‐free survival, whilst low levels are related to increased sensitivity to docetaxel, cisplatin and 5‐fluorouracil.^46^ Fn‐positivity is associated with chemotherapeutic resistance in CRC patients, where it is believed that activation of autophagic pathways promotes chemoresistance against both oxaliplatin and 5‐fluorouracil.^48^, ^49^ Modulation of chemotherapeutic resistance by Fn is likely mediated by the expression of IAP (Inhibitor of Apoptosis) protein BIRC3 (Baculoviral IAP Repeat Containing 3).^50^ BIRC3 and other IAP family proteins are well established in their role in mediating 5‐fluorouracil chemoresistance,^51^ thus the hypothesis that Fn induces increased BIRC3 expression to modulate chemosensitivity in CRC is logical.^50^ Recently, Fn has been shown to inhibit pyroptosis, the process usually responsible for mediating chemosensitivity, by inducing Hippo signalling in CRC cells. In this, upregulation of BCL2 occurs following Hippo activation, ultimately inhibiting the pyroptosis‐related pathway induced by chemotherapeutic agents.^52^

### 2.3 Metastasis

Fusobacterial tumour colonisation is heavily associated with poor patient outcomes, perhaps primarily attributable to the higher degree of disease progression and metastasis observed in these patients.^53^ Various mechanisms exist by which *Fusobacteria* can promote metastasis. Induction of chemokine expression by *Fusobacteria* mediates several metastatic processes. Expression of CCL20 by CRC cells is one such example, wherein *Fusobacteria* stimulates the NF‐κB signalling cascade, decreasing the expression of regulatory micro‐RNA‐1332 and facilitating overexpression on CCL20. Additionally, CCL20 encourages tumour microenvironment remodelling processes, which are essential to metastatic processes in many cancers.^53^ Moreover, upregulation of CXCL8 and CXCL1 by CRC cells (HT116) has been observed following Fusobacterial exposure and is associated with augmented metastatic potential in these cells.^54^ Comparatively, *Fusobacteria* has been shown to increase the expression of CXCL1 chemokine in prostate cancer. Binding of CXCL1 to CXC2 promotes metastasis in an autocrine manner, whereby the Akt pathway is activated to drive invasive behaviours.^55^ Fusobacterial‐induced chemokine release can also encourage a tumour‐permissive microenvironment. Following exposure to *Fusobacteria*, pancreatic cells increase expression of CXCL1,^56^ a chemoattractant pivotal in recruiting immune cells to tumour microenvironments. Recruitment of these immune cells, such as CXCR2‐positive myeloid‐derived suppressor cells, promotes the establishment of an immunosuppressed microenvironment by suppressing CD8+ T cells.^57^

*Fusobacteria* are also implicated in M2 macrophage polarisation, a process influential in metastasis. M2 polarisation of macrophages is attributed to chemoresistance and metastatic behaviour.^58^ M2 macrophages are traditionally considered ‘anti‐inflammatory’ compared to their M1 polarised counterparts. Expression of matrix metalloproteinases (MMPs) by M2 macrophages encourages the extracellular matrix remodelling, which enables the invasion and migration of malignant cells.^52^ CRC studies reveal that Fn induces M2 polarisation by activating the TLR4/NF‐κB/S100A9 cascade, wherein a proinflammatory microenvironment is established following S100A9 release to potentiate the ability of malignant colorectal cells to metastasise ultimately.^59^ Moreover, *Apc* ^Min/+^ mouse model studies show that this M2 polarisation process also occurs in vivo, wherein *Fusobacteria* elevate the presence of M2 macrophages. Importantly, pre‐treatment of mice with intraperitoneally injected TAK‐242 to block TLR4 attenuated macrophage polarisation suggesting that polarisation occurs in a TLR4‐dependent mechanism within colonic tumours.^60^

Induced expression of RNA by *Fusobacteria* may also encourage metastasis. Lung metastases derived from CRC can be mediated by long‐non‐coding RNA sequences such as Keratin7‐antisense (KRT7‐AS) and Keratin7 (KRT7), the expression of which is induced by Fn. KRT7‐AS depletion abrogates this metastasis, while upregulation encourages cellular invasion in HCT‐116 cells. NF‐κB signalling is believed to be responsible for this KRT7‐AS mediated metastasis, wherein inhibition of this cascade using BAY‐117082 significantly reduced upregulation of KRT7‐AS following Fusobacterial exposure.^61^ However, BAY‐117082 is not a specific NF‐κB inhibitor; instead, it has several anti‐inflammatory pharmacological applications which extend to a reduction in metastatic potential.^62^, ^63^ Expression of endogenous retroviral‐associated adenocarcinoma RNA (EVADR) is associated with decreased patient survival in colonic, lung and pancreatic cancers.^64^ In CRC, Fusobacterial‐mediated upregulation of EVADR correlates with increased metastatic potential across both in vitro and in vivo experimental models. Furthermore, EVADR amplifies epithelial‐to‐mesenchymal transition (EMT) by interacting with Y‐box binding protein 1 (YBX1) to encourage the translation of several downstream factors involved in EMT.^65^

## 3 FN‐HOST CELL INTERACTIONS

Given the vast heterogeneity of *Fusobacteria*, it is unsurprising that diversity exists regarding the expression of virulence factors, including adhesins. As the pathogenicity of *Fusobacteria* can be largely attributed to adhesin expression, it is predictable that these differences mediate, in part, the interaction with human cells. Predicting the exact number of adhesins and additional surface proteins expressed by members of the *Fusobacterium* genus is challenging, given the variability in the genome. Regardless, the role of these adhesins is well‐established, including biofilm formation, coaggregation and cellular adhesion.^66^, ^67^ For example, the expression of autotransporters (e.g. Fap2) on the Fusobacterial surface has been linked with coaggregation with Gram‐negative bacterial species such as *Porphyromonas gingivalis*, facilitating biofilm formation within the oral cavity.^68^ Many adhesive proteins expressed by *Fusobacteria* have been widely reported, and include Aid1 (adhesion inducing determinant 1) and CmpA (coaggregation mediating protein A),^69^ however, not all reported adhesins have been implicated in carcinogenesis. Hence, this review will focus on adhesins considered or evidenced as relevant to *Fusobacteria* in the context of the tumour microenvironment.

Importantly, differences in expression profiles have been observed in Fusobacterial adhesins relevant to carcinogenesis. The expression of one adhesin, FadA, was revealed to have at least 29 paralogues amongst the *F. nucleatum subsp. polymorphum* alone, illustrating significant heterogeneity.^70^ Furthermore, prominent homologues of FadA have also been identified in various Fusobacterial species, including *F. varium* and *F. ulcerans*.^71^ Similarly, FadA expression varies between Fn subspecies, further evidencing the complex and variable expression profiles of adhesins within *Fusobacteria*.^72^ Moreover, Fap2 homologues are lacking in *F. varium* but present in abundance within Fn and have an extensive influence on this species carcinogenic influence.^72^ Considering the variability in adhesin expression profiles, it is unsurprising that some Fusobacterial species are more associated with malignancy than others, such as the aforementioned *Fusobacterium nucleatum vincentii* and *Fusobacterium nucleatum animalis*.^20^

### 3.1 Fap2

Fap2 is a 390 kDa type Va autotransporter^73^ located in the outer membrane of *Fusobacterium*. This galactose‐binding adhesin contributes significantly to Fusobacterial virulence. In the oral cavity, Fap2 facilitates co‐adhesion with other pathogenic bacteria, such as *Porphyromonas gingivalis*.^73^ In a carcinogenic context, Fap2 directly binds the overexpressed polysaccharide tumactose‐β(1–3)‐N‐acetyl‐d‐galactosamine (Gal‐GalNAc), also known as the Thomsen‐Freidenreich antigen or CD176,^74^ on the surface of cancer cells. Typically, N‐acetylneuraminic acid, a major sialic acid, is added to the exposed ends of both GalNAc and Gal‐GalNAc residues.^75^, ^76^ However, this addition is often lacking in tumour cells, resulting in increased expression of these unmasked residues, which are implicated in malignant progression and invasion.^77^, ^78^ Interestingly, impaired cell binding ability has been observed in Fap2‐deficient *Fusobacteria* mutants,^73^ suggesting that Fap2 is essential for cancer cell‐specific binding. Further, recognising Gal‐GalNac subunits is necessary for tumour colonisation by Fusobacterial spp.^79^ Increased expression of Gal‐GalNAc moieties is observed throughout tumour progression.^80^, ^81^ In breast cancer samples, increased expression correlated with higher levels of internalised Fusobacterial gDNA and more extensive metastasis,^45^ potentially explaining the worsened prognosis observed in patients with higher tumoral Gal‐GalNAc expression.^81^

A potential downstream mechanism following Gal‐GalNAc binding by Fn may be through activation of the mitogen‐activated protein kinase (MAPK) signalling pathway.^82^ Dysregulated MAPK signalling is heavily implicated in carcinogenesis, with activation promoting cellular proliferation and apoptotic resistance^83^ Whilst the activation of MAPK signalling has not been directly linked with Fusobacterial Gal‐GalNAc adherence, in *Entamoeba histolytica*, activation of MAPK signalling following Gal‐GalNAc binding has been observed.^84^ Therefore, it is not unreasonable to suggest that Fn binding of Gal‐GalNAc via Fap2 may activate MAPK signalling in a similar process; however, further investigation is needed to explore this hypothesis.

**FIGURE 3:**
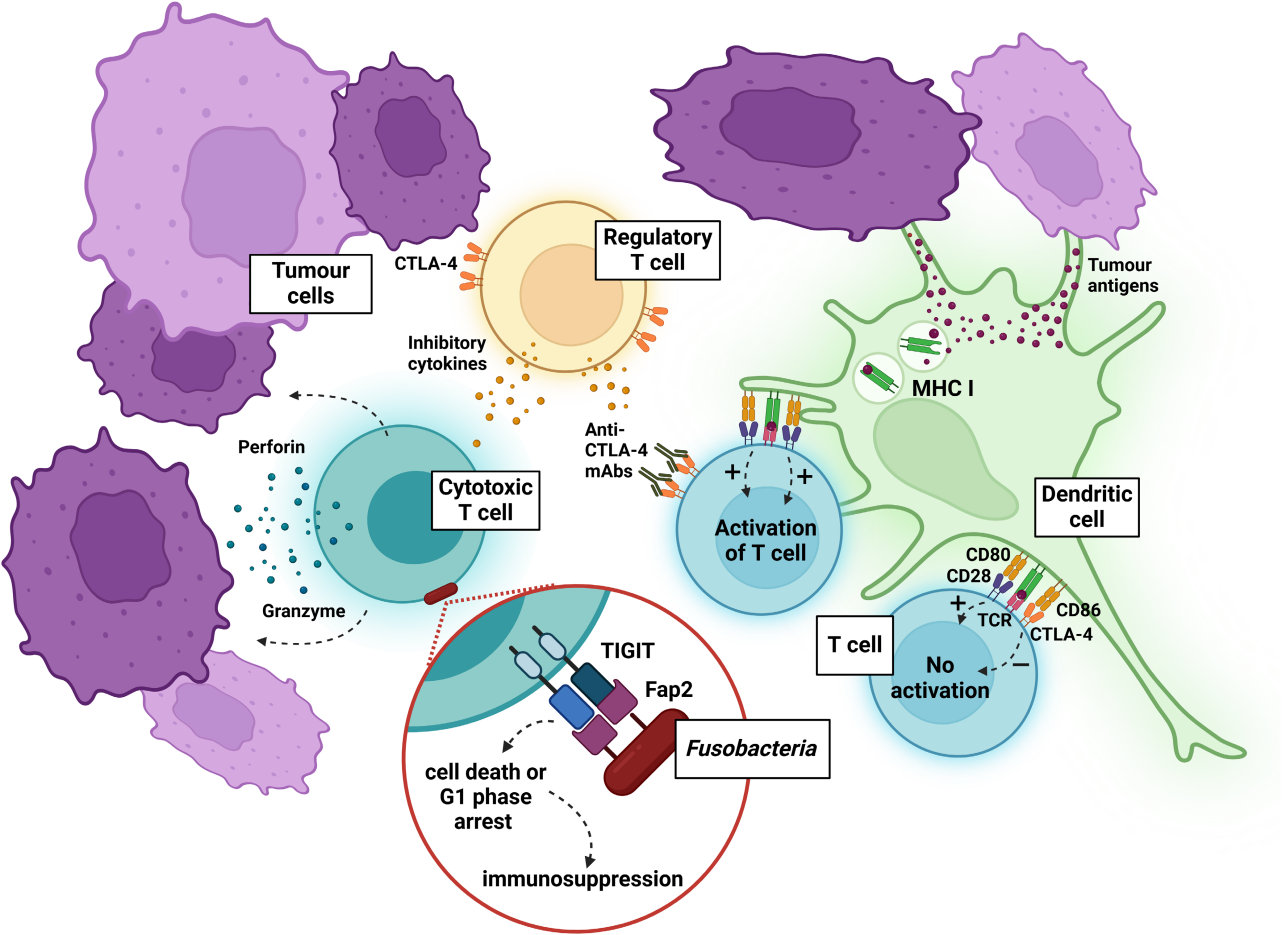
Anti‐tumoural immune responses. Components of both innate and adaptive immunity are involved in orchestrating anti‐tumoural immune responses. Dendritic cells form part of innate immunity, whilst cytotoxic and regulatory T cells are considered part of adaptive immunity. T cells become activated following the production of two activator signals, for example the binding of CTLA‐4 and anti‐CTLA‐4, or CD28 with CD80. Without two activator signals, T cells will not be activated, meaning that they do not enact any immune function. Together, cells of the immune system will coordinate anti‐tumoural immune responses to prevent proliferation of tumour cells (purple). *Fusobacteria* (red) can induce immunosuppression in many ways, including engaging with TIGIT domains on cytotoxic T cells via Fap2. Binding of TIGIT results in cell cycle phrase arrest in T cells, leading to immune suppression. Created in BioRender and adapted from information within.^86^

Aside from promoting a tumour‐permissive microenvironment, studies also suggest a role for *Fusobacteria* in dampening anti‐tumour immunity.^85^ One of the classical cancer hallmarks is immune evasion.^23^ Various components of both innate and adaptive immunity are involved in orchestrating these anti‐tumoral immune responses (Figure 3), including natural killer cells (NKs) and tumour‐infiltrating‐lymphocytes (TILs), respectively.^87^, ^88^, ^89^ Both NKs and TILs express T cell immunoreceptors with immunoglobulin G (IgG) and ITIM domains (TIGIT), which, when bound, dampens the immune response of these cells.^90^ Fusobacterial Fap2 has been observed to show TIGIT binding capacity,^85^ thereby providing a role for Fusobacterial‐mediated immune evasion. The binding of TIGIT by Fap2 attenuates the anti‐tumoural immune response by preventing NK cytotoxicity and inducing TIL cytotoxic cell death or G1 phase arrest.^91^ Direct TIGIT binding by Fap2 thus downregulates the anti‐tumour responses of both NKs and TILs. Moreover, TIGIT expression is upregulated in various tumour microenvironments—including colonic adenocarcinoma,^92^ non‐small cell lung cancer^93^ and acute myeloid leukaemia^94^—further facilitating the suppression of the anti‐tumoural immune response. Additionally, as TIGIT binding dampens immune responses against bacterial species,^95^ Fap2‐TIGIT binding is advantageous in allowing Fn to potentiate at tumour sites without immune detection. This establishes a vicious cycle of immune suppression, wherein Fap2‐TIGIT binding reduces both antibacterial and antitumoural immune responses, facilitating Fn growth and malignant cell immune evasion, respectively.

### 3.2 CbpF

**FIGURE 4:**
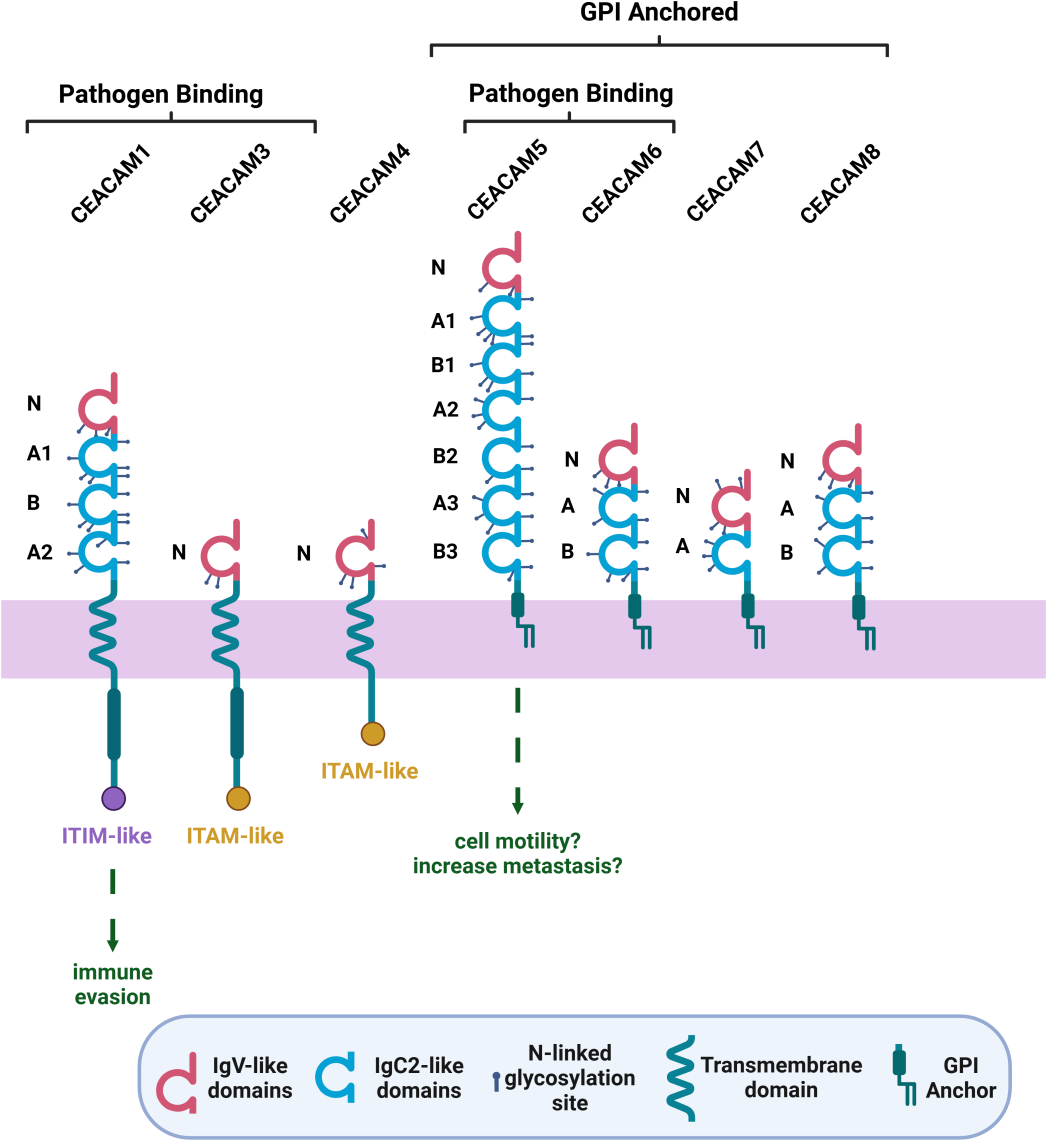
CEACAM structure schematic. The structure of eight of the known 12 human CEACAM proteins is displayed, with the Ig domains detailed to the left of each schematic. Both ITIM‐like (immunoreceptor tyrosine‐based inhibitory motif) and ITAM‐like (immunoreceptor tyrosine‐based activation motif) motifs are displayed. The downstream effects of Fusobacterial binding are detailed in green. Adapted from^98^, ^99^ and created using BioRender.

**FIGURE 5:**
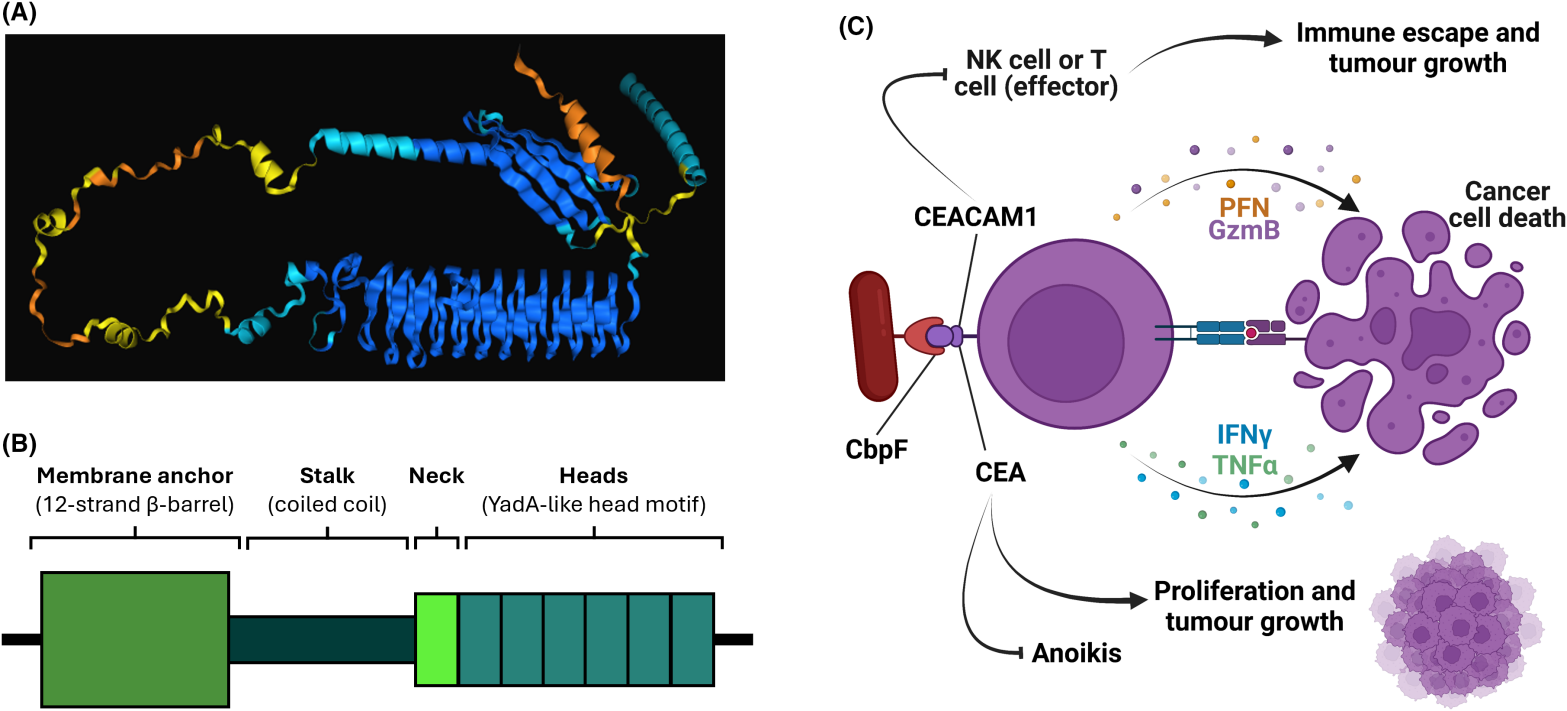
CbpF. (A) An AlphaFold predicted structure is depicted (unpublished data). (B) A simplified schematic of the CbpF structure. The trimeric autotransporter contains four major domains. The membrane anchoring domain exists as a 12‐strand β‐barrel, whilst the stalk of CbpF likely exists as a coiled‐coil structure. The neck and head domains follow, with each of the six head domains likely adopting a YadA‐like head motif. Adapted from.^21^ (C) The downstream impacts of CbpF of CEACAM1/CEA binding are shown. Image created in BioRender.

Additional immune inhibitory roles for *Fusobacteria* exist. *Fusobacteria* can bind an alternative immune inhibitory receptor within the immunoglobulin superfamily known as carcinoembryonic antigen‐related cell adhesion molecule 1 (CEACAM1).^96^ The structure of CEACAM1 is unique in that it contains an ITIM (immunoreceptor tyrosine‐based inhibitory motif) domain^97^ alongside the typical extracellular N‐terminal domain and up to three immunoglobulin‐like domains (Figure 4) as seen in other CEACAM family proteins.^96^ The binding of CEACAM1 is facilitated by CbpF (Figure 5), a type Vc trimeric autotransporter that is expressed in two forms: CbpFa (144 kDa) and CbpFb (154 kDa).^21^ Similarly to TIGIT, as discussed above, CEACAM1 is also expressed on the surface of NKs and TILs.^100^, ^101^ The expression of CEACAM1 by CD4+ TILs is associated with decreased interferon‐ γ (IFNγ), a marker of T cell exhaustion. This suggests that CEACAM1 plays a significant role in mediating T‐cell exhaustion.^102^, ^103^ CEACAM1 expression is only detectable in active NKs^99^. Following IL‐2 exposure, the expression of CEACAM1 is upregulated, leading to impaired natural killer gene 2 member D (NKG2D)‐induced cytolysis. NKG2D engagement typically leads to cytolysis of target cells,^104^ except when both NKs and target cells (e.g. tumour cells) express CEACAM1. Here, the homophilic interaction of CEACAM1 displays an inhibitory effect, preventing NK‐mediated tumour killing and, therefore, permitting tumour growth.^105^ The direct binding of CbpF to CEACAM1 provides an alternative to this homophilic binding. Still, it retains the inhibitory capacity, further dampening the anti‐tumoural immune response and creating a tumour‐permissive environment.^102^

Malignant cells also express CEACAM1 on their surface.^106^ Expression is observed in multiple tumour types and correlates with cancer progression and metastasis.^107^, ^108^, ^109^ This provides homophilic binding to the CEACAM1, also expressed on NKs and TILs, facilitating immune evasion by malignant cells.^110^ The binding of Fusobacterial CbpF to CEACAM1 expressed on malignant cells has been observed in CRC.^21^ Interestingly, *H. pylori*, the only evidenced carcinogenic bacterium, also possesses CEACAM1 binding capacity via its HopQ adhesin^111^ with Downstream activation of canonical and non‐canonical NF‐κB pathways are observed following HopQ‐CEACAM1 binding,^112^ however, this effect has yet to be shown regarding CbpF‐CEACAM1 binding.

Additionally, CbpF has been shown to interact with the tumour‐related protein CEA,^97^ encoded by the gene *CEACAM5*.^102^ The expression of CEA is found in healthy and malignant tissues. However, expression significantly increases in cancer cells with diverse roles in malignant behaviour. Like other CEACAM proteins, CEA promotes the proliferation and adhesion of malignant cells. Still, it has also been shown to inhibit anoikis^113^ and promote metastasis of non‐small cell lung cancer^114^ and liver cancer.^99^ The exact role of CbpF binding to CEA in promoting metastasis is yet to be elucidated. Therefore, future research efforts should seek to uncover this adhesin‐ligand relationship regarding carcinogenesis.

### 3.3 FadA

**FIGURE 6:**
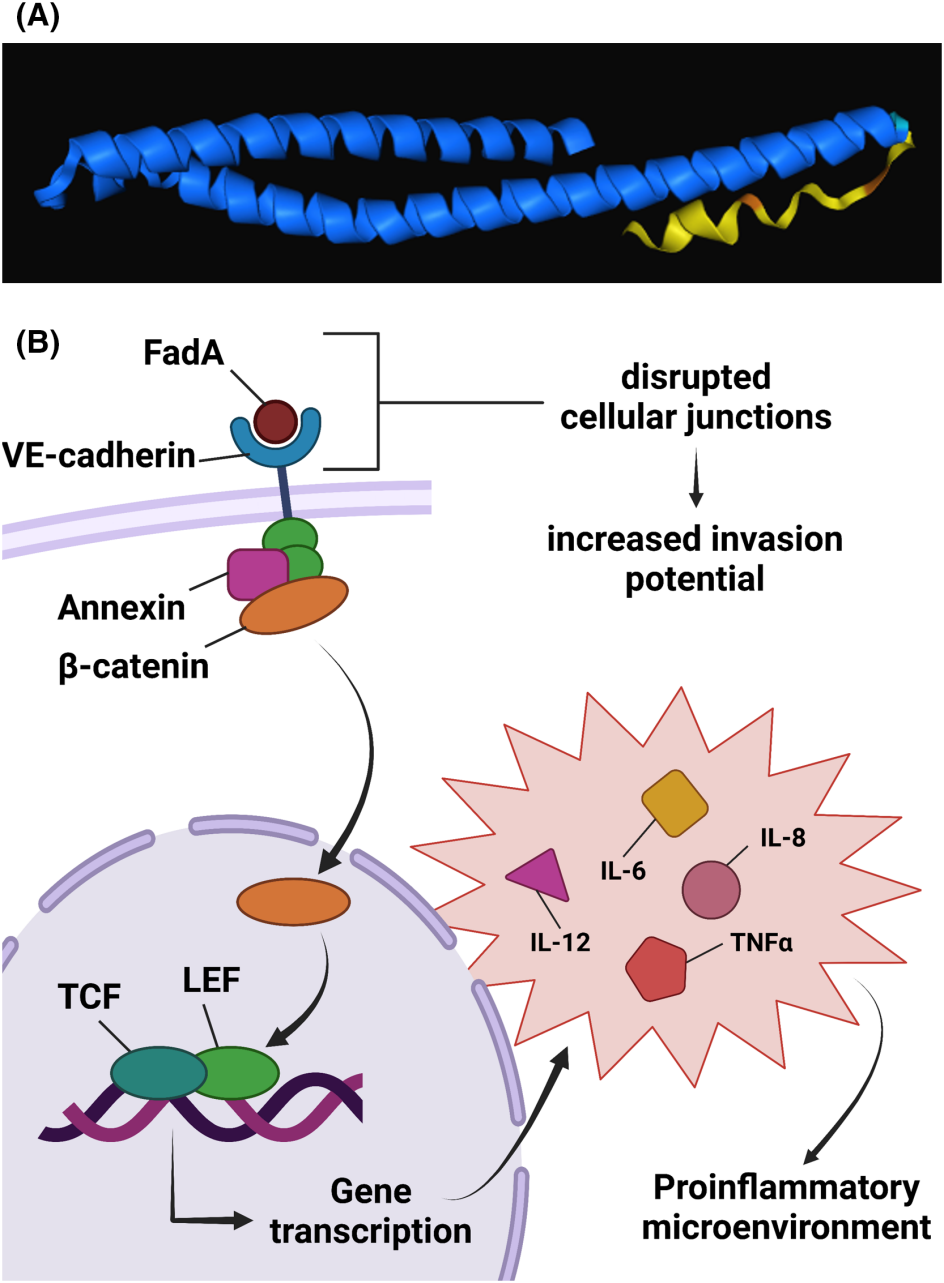
FadA. (A) AlphaFold predicted FadA structure (unpublished data). (B) The associated downstream mechanisms involved in the binding of FadA to VE‐Cadherin. Transcription of genes leads to the expression of proinflammatory cytokines, which in turn adds to the tumour permissive microenvironment. Image created using BioRender.

FadA is an outer membrane protein with a small α‐helical structure that is highly conserved in oral *Fusobacteria* and exists in both secreted form (12.6 kDa) and intact form (13.6 kDa).^115^, ^116^ Membrane‐anchored, intact, FadA—otherwise termed preFadA—consists of 129 amino acid residues, whilst the secreted, mature FadA (mFadA) consists of only 111 amino acids, with mFadA being responsible for the adhesin's function.^117^ Interactions between *Fusobacteria* and other bacterial species are mediated by FadA, including *P. gingivalis*, *S. oralis*, *A. naeslundii* and *S. gordonii*.^118^, ^119^ Aside from roles in bacterial coaggregation, FadA can also interact with human cells. FadA binds vascular endothelial cadherin (VE‐cadherin) (Figure 6). The structure of VE‐cadherin is divided into five extracellular domains, a conserved intracellular domain and one single transmembrane domain.^120^ FadA interacts most prominently in the region which spans the end of the fourth and beginning of the fifth extracellular domains (residues 415–534).^121^ FadA is essential to the adherence and invasion of host cells in vitro by Fn.^122^ Notably, VE‐cadherin is expressed by endothelial cells and various types of malignant cells, including colorectal and breast,^123^ meaning that Fn can bind multiple cell types using FadA. The binding of VE‐cadherin by FadA promotes Wnt/β‐catenin signalling in cells, ultimately promoting a proinflammatory microenvironment.^121^, ^124^ In the Wnt signalling cascade, Wnt binds Frizzled/LRP to inhibit the degradation of β‐catenin, thereby allowing transcriptional activation of Wnt target genes that ultimately drive cellular proliferation.^125^, ^126^ Moreover, Wnt signalling can participate in proinflammatory processes by the upregulation of NF‐κB and scavenger receptor A,^127^ enabling proinflammatory cytokine expression; TNF, IL‐6 and IL‐12.^128^ Thereby, stimulation of Wnt/β‐catenin signalling by FadA‐VE cadherin binding adds to the pro‐carcinogenic role of *Fusobacteria*.

Typically, VE‐cadherin is distributed in intercellular junctions only,^129^, ^130^ with significant roles in maintaining vascular integrity.^131^ Interestingly, the binding of VE‐cadherin by FadA alters the distribution of this adhesion molecule, resulting in VE‐cadherin presence in cytoplasmic compartments and at cellular junctions.^121^ This internalisation results in loosened cell–cell junctions, allowing enhanced cell migration.^132^, ^133^, ^134^ In this, the binding of FadA to VE‐cadherin may facilitate increased migration of malignant cells, thereby allowing the increased invasion of tumours into neighbouring tissues and enhancing metastatic potential.

Interrupting the interaction between FadA and VE‐cadherin may provide an avenue for therapeutic intervention regarding Fusobacterial presence in tumours. The use of phytochemicals in herbal cancer medicine is well documented,^135^, ^136^, ^137^ with studies suggesting synergism with conventional chemotherapeutic agents.^138^, ^139^ Phytochemicals can also display antimicrobial activity.^140^ Investigation into the applications of phytochemicals for *Fusobatcerial* colonisation of colorectal tumours has revealed the antimicrobial potential of sodium new houttuyfonate (SNH)^141^: a chemical derived from the common Chinese medicine plant *Houttuynia cordata*.^142^ Whilst SNH showed little cytotoxicity against malignant colorectal cell lines (HCT116 and HT29); however, it did display antimicrobial activity against Fn.^141^ It is thought that SNH disrupts the inner and outer bacterial membranes to exhibit this antimicrobial activity.^141^ Importantly, SNH also displays antimicrobial activity against other Gram‐negative bacteria, such as *Pseudomonas aeruginosa*,^142^ as well as Gram‐positive bacteria, including *Staphylococcus aureus, Streptococcus pneumoniae*, and *Streptococcus mutans*.^143^, ^144^, ^145^ FadA is the proposed target of SNH, suggesting that SNH binding results in oligomerisation and disruption of membrane integrity.^141^ Considering that FadA is exclusive to *Fusobacterium*,^115^ it is reasonable to suggest that SNH may provide therapeutic intervention for Fn‐mediated CRC progression.

Alternative methods for counteracting Fn‐mediated cancer progression may lie in using antimicrobial peptides. These bioactive molecules have garnered increasing attention in recent years due to their low toxicity and low risk of resistance development.^146^, ^147^ Jelleine‐I (J‐I) has been observed to suppress Fn‐mediate CRC progression.^148^ Isolated initially from honeybees' royal jelly,^149^ J‐I exhibits antibacterial and antifungal properties.^150^ Concerning CRC, halogenated J‐I analogues have shown potential in combating the pro‐tumourigenic impacts of *Fusobacteria*.^148^, ^151^ Of these, bromine‐J‐I (Br‐J‐I) was noted to exert the highest antibacterial activity against Fn, inhibiting both Fn‐induced inflammation and CRC progression.^148^ Similarly to SNH, described above, Br‐J‐I is also suggested to interact with FadA. It is proposed that Br‐J‐I binds to the oligomerisation region of FadA, fitting within the amphipathic groove. Br‐J‐I triggers FadA oligomerisation in this, ultimately resulting in a permeabilised membrane.^148^ Following co‐culture with Br‐J‐I, the level of Fn‐induced cellular proliferation was suppressed, suggesting that Br‐J‐I may provide an avenue to reduce Fn‐mediated tumour growth. Moreover, Br‐J‐I reduced the degree of Fn‐mediated inflammation.^148^ These results suggest that Br‐J‐I may have a therapeutic application in reducing the procarcinogenic effects exerted by Fusobacterial spp.

### 3.4 FomA

**FIGURE 7:**
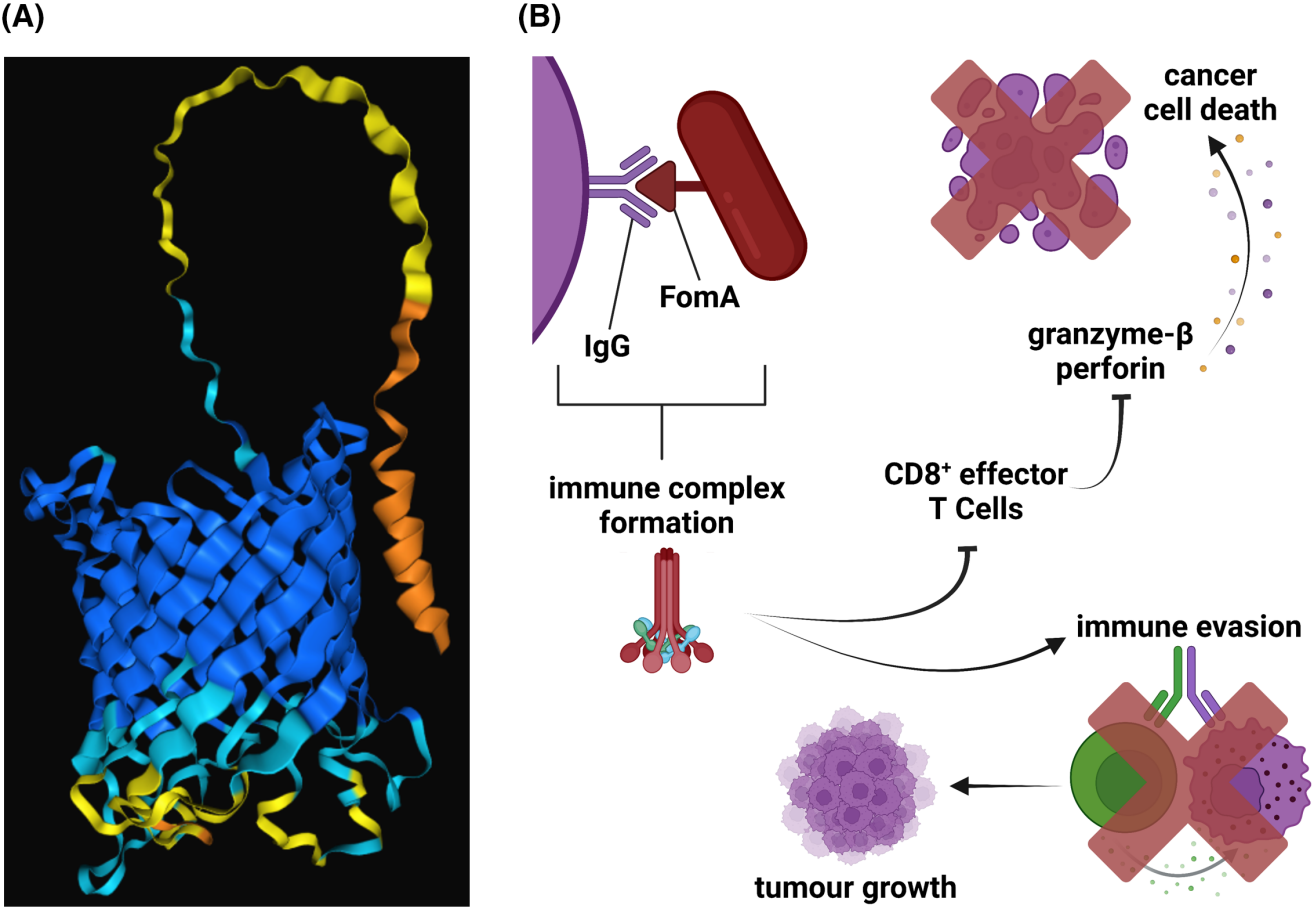
FomA. (A) AlphaFold predicted FomA structure (unpublished data). (B) The associated downstream mechanisms involved in the binding of FomA to IgG. Immune suppression following this binding facilitates tumour growth. Image created using BioRender.

Aside from Fap2, FomA (Fusobacterial outer membrane protein A) is the other major outer membrane protein of *Fusobacterium* ^152^ (Figure 7). FomA plays a pivotal role in biofilm formation, facilitating coaggregation with other pathogenic bacteria such as *P. gingivalis*: a process that is heavily implicated in the role of *Fusobacteria* in periodontal diseases.^153^, ^154^, ^155^ FomA, a voltage‐dependent non‐specific porin, also can bind the Fc portion—constant domain—of human immunoglobulin G (IgG).^156^ This activity has been demonstrated in multiple Fn strains, including ATCC 25586.^157^ Elevated IgG levels have been observed in various tumour types.^158^ IgG serum levels are prominently elevated in cancerous and precancerous patients with oral malignancies,^159^ with the presence of *Fusobacteria* possibly being the reason for this increased IgG secretion by cancerous cells.^158^ This elevated expression of secreted IgG is pronounced in CRC compared to healthy tissues.^160^, ^161^ Thus, there are more binding opportunities for FomA in cancers, allowing for augmented colonisation by *Fusobacterium*.

The binding and subsequent activation of IgG by FomA has several implications in carcinogenesis. Increased IgG expression by CRC cells correlates with several aggressive clinicopathological features, including lymph node involvement, inflammatory infiltration and TNM (tumour, node, metastasis) stage.^161^ Interestingly, coexpression of IgG with CEA has been observed in CRC tissue samples, giving further interaction opportunities between cancerous cells and *Fusobacteria* in the form of aforementioned Fap2‐CEA binding. Moreover, the intensity of IgG‐positive staining is directly linked with CRC grade, with well‐differentiated adenocarcinomas displaying the lowest degree of positive immunohistochemical staining.^161^ IgG expression also correlates with increased aggressive cellular behaviour in other cancers, including breast,^162^ prostate,^163^ sarcoma^164^ and bladder.^165^

Elevated IgG levels are associated with increased tumour size and the chance of recurrence in bladder cancer patients.^165^ Tumour size and recurrence rate are intimately correlated with cancer survival rates, meaning that elevated IgG levels are associated with a worsened prognosis.^166^, ^167^, ^168^ In breast cancer cells, IgG expression is associated with increased proliferation rates and colocalisation of C1q complement, suggesting the formation of immune complexes.^162^ Following binding of the Fc portion of IgG (e.g. by FomA of *Fusobacterium*), C1q is recruited to initiate the membrane attack complex (MAC).^169^, ^170^ The MAC then initiates lytic destruction of the target cell by damaging cell membrane integrity, capable of orchestrating both host‐cell (e.g. tumour) and bacteriocidal killing.^171^, ^172^ Immune complexes are formed when multivalent antibodies bind to multiple antigens with high avidity. In cancerous patients, these IgG‐based immune complexes are more prominent than their healthy counterparts.^173^, ^174^ Exposure of CD8^+^ T cells to IgG immune complexes displays inhibitory activity in murine models.^175^ However, in human models, this suppressive role has not been observed. Instead, IgG immune complexes have been observed to diminish the expression of granzyme‐β and perforin by cytotoxic memory T cells (CD8^+^).^176^ Furthermore, the inhibition of naïve T cells is also observed following exposure to IgG immune complexes.^176^ In this, the binding of FomA to IgG promotes the formation of immune complexes, thereby attenuating both naïve and effector T cell functions. Thus, the interaction between FomA and the Fc portion of IgG provides further evidence for the role of *Fusobacteria* in facilitating immune evasion of malignant cells to ultimately encourage carcinogenesis.

The existence of a FomA‐based vaccine may provide therapeutic intervention in cancer patients, particularly CRC. Directed against FomA, these Fn‐directed vaccines were designed to prevent oral pathologies associated with *Fusobacteria*, such as oral abscesses formation and halitosis.^177^ Further FomA‐based vaccine research revealed that targeting this outer membrane protein could successfully abrogate bacterial coaggregation and, thus, biofilm formation in oral cavity models.^178^ Effective targeting of FomA has demonstrated significant capacity in preclinical models to reduce the ability of *Fusobacteria* to induce periodontal disease.^179^, ^180^ Whilst using a FomA‐based vaccine has not yet been explored in cancer models, it may provide therapeutic benefits to patients. However, it is essential to note that this notion is purely speculative^181^ and that the incidence of CRC, or other *Fusobacteria*‐mediated cancers, following FomA‐based vaccines is lacking in data. Nonetheless, future research may wish to explore the effects of FomA‐based vaccines in cancer models.

## 4 CONCLUSION

The role of Fusobacterial spp. in tumour development and progression is diverse. The Fusobacterial interactome is evidenced to promote several aspects of carcinogenesis. Fusobacterial outer membrane proteins promote several aspects of a tumour‐permissive microenvironment, including dampening anti‐tumour immunity via Fap2‐TIGIT and CbpF‐CEACAM‐5 interactions. Furthermore, Fusobacterial binding can drive cell signalling processes implicated in several cancers, such as Wnt/β‐catenin and MAPK signalling. However, many questions remain unanswered. Further characterisation of additional adhesins and receptors will likely further evidence the pro‐carcinogenic role of *Fusobacteria*. Given the amassing literature which suggests that Fn may also be implicated in other malignancies, future research should prioritise the inclusion of various cell lines to consider a range of cancers, including breast and ovarian. Considering the limitations of traditional 2D culture of cell lines, future research may wish to employ 3D models such as organoids to recapitulate better human physiology, which would be invaluable in exploring potential therapeutic interventions. Future research should also seek to understand better the relationship between *Fusobacteria* and the intracellular signalling pathways at play within malignant cells, using a variety of genetic modification approaches and transgenic mouse models for example, CEACAM1 transgenic mice, to elucidate these impacts. In this, research efforts may uncover further evidence for the role of *Fusobacteria* in carcinogenesis and identify areas of potential therapeutic intervention regarding the Fusobacterial colonisation of tumours.

## CONFLICT OF INTEREST STATEMENT

The authors have no conflicts of interest to declare relating to this article.

## Data Availability

Data sharing not applicable to this article as no datasets were generated or analysed during the current study.
